# Reliability and accuracy of EEG interpretation for estimating age in preterm infants

**DOI:** 10.1002/acn3.51132

**Published:** 2020-08-07

**Authors:** Nathan J. Stevenson, Maria‐Luisa Tataranno, Anna Kaminska, Elena Pavlidis, Robert R. Clancy, Elke Griesmaier, James A. Roberts, Katrin Klebermass‐Schrehof, Sampsa Vanhatalo

**Affiliations:** ^1^ Brain Modelling Group QIMR Berghofer Medical Research Institute Brisbane QLD Australia; ^2^ Department of Neonatology Wilhelmina Children’s Hospital University Medical Center Utrecht Utrecht The Netherlands; ^3^ Department of Clinical Neurophysiology Necker‐Enfants Malades Hospital APHP Paris France; ^4^ INSERM U 1141 Neurodiderot Paris France; ^5^ Child Neuropsychiatry Service of Carpi Mental Health Department AUSL Modena Carpi Italy; ^6^ Department of Pediatrics (Neurology) Children's Hospital of Philadelphia Philadelphia Pennsylvania; ^7^ Department of Pediatrics (Neonatology) Medical University of Innsbruck Innsbruck Austria; ^8^ Department of Pediatrics and Adolescent Medicine Division of Neonatology Pediatric Intensive Care and Neuropediatrics Medical University of Vienna Vienna Austria; ^9^ BABA Center Department of Clinical Neurophysiology Children’s Hospital Helsinki University Hospital University of Helsinki Helsinki Finland; ^10^ Neuroscience Center Helsinki Institute of Life Science University of Helsinki Helsinki Finland

## Abstract

**Objectives:**

To determine the accuracy of, and agreement among, EEG and aEEG readers’ estimation of maturity and a novel computational measure of functional brain age (FBA) in preterm infants.

**Methods:**

Seven experts estimated the postmenstrual ages (PMA) in a cohort of recordings from preterm infants using cloud‐based review software. The FBA was calculated using a machine learning‐based algorithm. Error analysis was used to determine the accuracy of PMA assessments and intraclass correlation (ICC) was used to assess agreement between experts.

**Results:**

EEG recordings from a PMA range 25 to 38 weeks were successfully interpreted. In 179 recordings from 62 infants interpreted by all human readers, there was moderate agreement between experts (aEEG ICC = 0.724; 95%CI:0.658–0.781 and EEG ICC = 0.517; 95%CI:0.311–0.664). In 149 recordings from 61 infants interpreted by all human readers and the FBA algorithm, random and systematic errors in visual interpretation of PMA were significantly higher than the computational FBA estimate. Tracking of maturation in individual infants showed stable FBA trajectories, but the trajectories of the experts’ PMA estimate were more likely to be obscured by random errors. The accuracy of visual interpretation of PMA estimation was compromised by neurodevelopmental outcome for both aEEG and EEG review.

**Interpretation:**

Visual assessment of infant maturity is possible from the EEG or aEEG, with an average of human experts providing the highest accuracy. Tracking PMA of individual infants was hampered by errors in experts’ estimates. FBA provided the most accurate maturity assessment and has potential as a biomarker of early outcome.

## Introduction

Scalp recorded electroencephalography (EEG) is widely used for bedside assessment of newborn brain function. It is often used for monitoring epileptic seizures, and to track functional brain recovery after medical adversities such as birth asphyxia or early preterm birth.^1^, ^2^, ^3^, ^4^, ^5^, ^6^ Recent advances in neonatal intensive care unit (NICU) treatment protocols have emphasized the need for improved, evidence‐based measures of functional brain development. Several studies have indicated that early adversities or compromised neurodevelopment are associated with a developmental delay in neonatal EEG.^4^, ^7^, ^8^, ^9^, ^10^, ^11^, ^12^, ^13^ EEG would, therefore, be an ideal tool for tracking functional brain maturation in both research trials and clinical routine. The visual interpretation of the EEG is, however, subjective and its general applicability is necessarily predicated on its reliability.

Clinical evaluation of the neonatal EEG is based on visual interpretation of the raw signal and/or its compressed version, for example, the amplitude integrated EEG (aEEG).^14^ Newborn EEG activity undergoes rapid developmental changes during the early weeks of life and a clinical EEG assessment is expected to determine the relative maturity of the EEG activity compared to an infant’s postmenstrual age (PMA).^7^, ^15^, ^16^, ^17^ Given the importance of EEG for assessing brain function in preterm infants, it is notable how little data exists on the accuracy of an expert’s visual assessment of EEG maturity.^18^, ^19^ Clinical practice is based on the common belief that an experienced EEG reader would be able to assess EEG maturity with an accuracy of 2 weeks.^15^

The aims of this study were to measure the accuracy and reliability of clinical expert readers for assessing brain maturity in EEG and aEEG recordings from a cohort of preterm infants and compare visual review to a novel, computational measure of functional brain age (FBA).

## Methods

**Figure 1:**
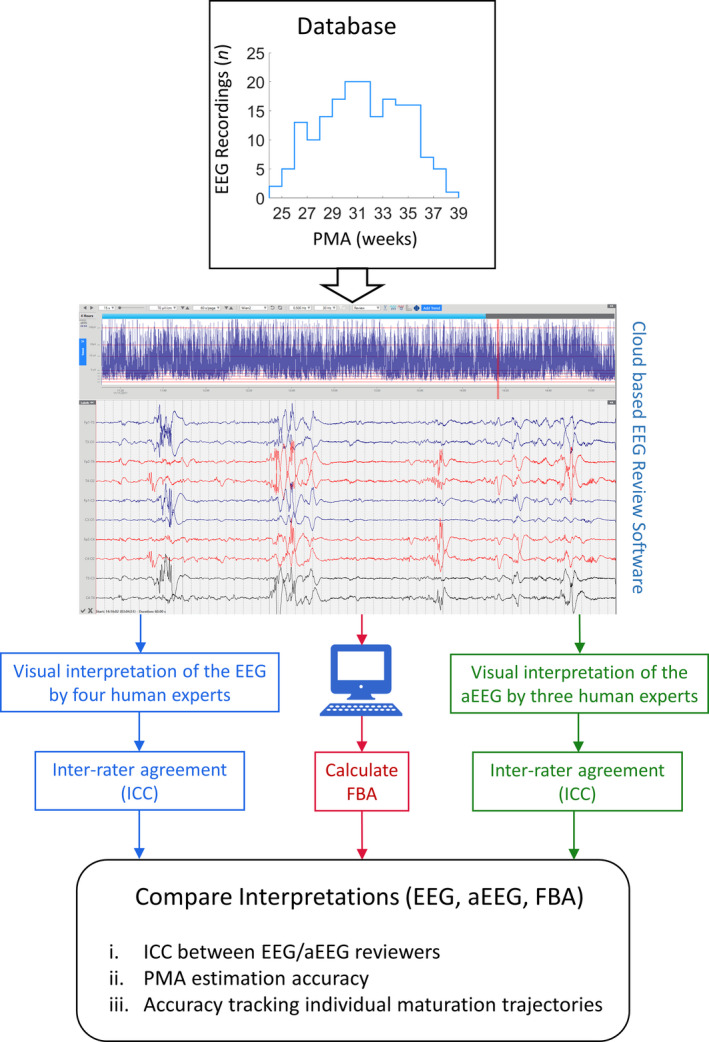
Study overview: this figure depicts a display of 4 h of aEEG and 60 sec of conventional EEG, recorded from an infant at a PMA of 26 weeks.

An overview of the study is shown in Figure 1.

### Data acquisition

Two hundred and thirty‐two (232) EEGs were recorded from a cohort of 67 preterm infants consecutively admitted to the NICU of the Medical University Hospital of Vienna between October 2011 and July 2015. The median recording duration was 2.7 h (IQR: 2.0–3.1 h, min: 0.4 h, max: 7.8 h) and the median number of recordings per infant was 4 (IQR: 3–4, min: 1, max 5). Initial tracings were obtained as soon as possible after birth and consecutively at 2‐week intervals until term equivalent age, when possible (range: 25–38 weeks PMA). Written, informed parental consent was obtained from the parents. A subset of this cohort was previously used to develop computational methods of EEG analysis.^13^, ^20^ Infants were included in the study cohort if they were born before 28 weeks gestational age (GA) and they were medically stable at the time of EEG recordings.

EEG recordings were made using nine scalp electrodes and a Brain Quick/ICU EEG (MicroMed, Treviso, Italy) at a sampling frequency of 256 Hz. Electrode positions employed the international 10–20 system modified for neonates at Fp1, Fp2, C3, C4, T3, T4, O1, O2, with a reference at Cz. The default EEG montage for conventional EEG review was Fp1‐T3, T3‐O1, Fp2‐T4, T4‐O2, Fp1‐C3, C3‐O1, Fp2‐C4, C4‐O2, T3‐C3, C4‐T4. The default aEEG montage was C3‐C4.

### Visual EEG and aEEG review

Seven clinical, neonatal EEG expert readers from six countries reviewed the recordings. Four reviewed the EEG signal (AK, EP, RC, SV) and three reviewed the aEEGs (EG, KKS, MLT). All reviewers were trained under different mentors and recording environments (Austria, Finland, France, Italy, Netherlands, USA) and all are internationally recognized experts with 7–30 years (mean 16 years) reading experience. Hence, we expected the assessments of accuracy and reliability to represent the upper end of the range seen in routine clinical practice.

All human experts reviewed the same EEG files using the same online review software. This was made possible by using a clinically approved cloud‐based EEG platform where each EEG/aEEG reviewer accessed the recording database using a local installation of the software (iEEG/Stratus, version 2.2.5253.13840, Kvikna Medical ehf., Iceland). Reviewers were able to alter the montage, filter settings, time and voltage scaling as per clinical practice. To ensure full blinding, the EEG files were converted to EDF, renamed using randomized codes, and uploaded onto the online review platform.

Reviewers were asked to estimate the PMA of the infant for each EEG file. Except for the broad age range of the cohort (25–38 weeks PMA), no other information or video about the infants was disclosed. Reviewers were not required to explain their estimates, but comments were collected on any recording issues that could interfere with PMA assessments, such as limited recording length, excessive artefact, or the presence of pathological EEG/aEEG patterns. Reviewers were encouraged to give PMA estimates in all cases where possible, irrespective of concerns about technical quality or pathological findings. EEG recordings were excluded from further analysis if: (1) at least two out of seven reviewers commented on excessive artefact in the recording, (2) if at least two reviewers commented that the recording was too brief for analysis, or (3) missing age estimates from at least one reviewer.

### Computational estimate of functional brain age (FBA)

FBA was calculated by a recently developed machine learning‐based algorithm that estimates maturation based on a multivariable regression model with quantitative EEG (qEEG) variables as inputs.^13^ The FBA algorithm forms an estimate of age using a combination of computational summary measures of 1 h epochs of EEG recordings. These computational measures of EEG were designed to (1) reflect the visual interpretation of EEG with measures such as amplitude, inter‐burst interval, and relative frequency band powers, and (2) reflect several signal measures that are not explicitly observed in visual interpretation such as entropy, suppression curve, the distribution of bursts versus their durations, and average shapes of bursts normalized over time and amplitude.^13^ The FBA algorithm also has initial pre‐processing stages to remove suspected periods of artefact based on criteria for excessive amplitude. The function that combines these features into an estimate of age was estimated using support vector regression trained on subsets of data used in this study. FBA used in this study was evaluated on the current dataset using leave‐one‐out cross validation.^21^ For EEG recordings that were longer than 1 h in duration, the average FBA over multiple 1 h epochs was used as the measure of FBA.

### Statistical analysis

Inter‐rater agreement among expert readers was evaluated using the intra‐class correlation coefficient (ICC) which assumes that “a random sample of *k* judges is selected from a larger population, and each judge rates each target, that is, each judge rates *n* targets altogether”.^22^ The age estimates (*k* = 4 for the EEG, *k* = 3 for the aEEG) were compared across EEG recordings.

Inter‐rater agreement between EEG and aEEG estimates of PMA was compared using resampling methods (bootstrap), i.e. the distributions were calculated for the differences between ICC of aEEG and EEG estimates of PMA for 1000 resampled subsets. The 95%CI was used to determine a significant difference in ICC, that is, if the 95%CI did not span zero then the ICC was deemed to be significantly different. Systematic differences between individual EEG and aEEG reviewers were assessed using the Kruskal–Wallis test. The reviewers’ assessments were also compared on an individual by individual basis with Pearson’s linear correlation coefficient. Bootstrap resampling was used for estimating confidence intervals of the correlation coefficients.

The accuracy of age estimation was evaluated using two forms of error: Systematic error (bias or average deviation from the PMA) and random error (variance or point‐wise deviation from the PMA). Pearson’s correlation coefficient and standard deviations were used to measure random error and assessed for individual interpretations and for an ensemble interpretation (average across all reviewers). These measures were supported by additional stratified measures of accuracy such as the percentage of recordings within 1 week and 2 weeks of PMA. The ensemble age estimate was also compared to a computational estimate of age (FBA). For comparisons including the FBA, we used only EEG recordings that were successfully annotated by both human expert reviewers and the computer algorithm (*n* = 149), the latter of which includes a separate, automated artefact rejection stage. Differences in the mean error (systematic error) between the PMA and the visual interpretation of age or FBA were tested using t‐tests (paired data) or Welch’s *t*‐test (unpaired data). Differences in the error standard deviation (random error) between the PMA and the visual interpretation of age or FBA were tested using the Pitman Morgan test (paired data) or Barlett test (unpaired data).

The ability of visual interpretation to track trajectories of brain maturation across consecutive EEG recordings was evaluated by computing the standard deviation of the difference between estimated age and PMA per infant. Only infants with at least two recordings were included (*n* = 47). The difference in infant‐wise standard deviations between interpretations (EEG, aEEG, FBA) was evaluated using a one‐way ANOVA. For the ANOVA, the homogeneity of group variances was tested using Levene’s absolute test and post‐hoc analysis was performed using the Tukey’s HSD test. The standard deviation variable was log‐transformed for the ANOVA.

The effects of pathologic conditions on ICC and PMA estimation accuracy were also examined. Three definitions of abnormality were used: (1) Visual EEG interpretation, whereby a recording was deemed abnormal if at least one reviewer annotated a specific EEG abnormality such as low voltage activity, absent sleep‐wake cycling, sharp waves, dysmorphic delta brushes or burst suppression; (2) Neuropathological lesions, whereby an EEG was recorded from an infant with a diagnosis of IVH or PVL; (3) Neurodevelopmental abnormalities, whereby an EEG was recorded from an infant with subsequent scores on the Bayley Scales of Infant Development more than two standard deviations below the population average (less than 70) in any domain. PMA estimates were grouped into normal or abnormal groups, differences in ICC were compared using a bootstrap analysis and differences in PMA accuracy were evaluated using Welch’s *t*‐test (testing systematic error) and the Bartlett test (testing random error).

## Results

As detailed in Table S1, 13 infants developed an IVH (grade 2 or 3), one infant developed PVL (grade 2), two infants developed NEC, and 18 infants had chronic lung disease. While recording the first EEG, eight infants received sedative analgesic or anti‐seizure medications. While recording the second EEG, four infants received such medications. While recording the third EEG, two infants received such medications and while recording the fourth EEG, one infant received such medications. No infants were on these medications during the fifth and sixth EEG.

Visual interpretations were obtained on 179 recordings from 62 infants using the full EEG or aEEG. This cohort was used to evaluate inter‐rater agreement using the ICC (ICC cohort). Automated FBA measures were computed on 177 recordings from 65 infants. A total of 146 recordings from 61 infants were interpreted by both human experts and FBA calculations (the FBA algorithm has internal artefact detection, see Figs. S1–S4). This cohort was used to evaluate the accuracy of age estimation (PMA cohort). A summary of clinical variables within both cohorts is shown in Table S1. Finally, the accuracy in tracking maturation of individual infants was evaluated on a subset of infants from the PMA cohort with serial EEG recordings.

### Comparison of EEG and aEEG interpretations

The ICC between EEG reviewers was 0.517 (95%CI: 0.311–0.664; *n* = 179), and the ICC between aEEG reviewers was 0.724 (95%CI: 0.658–0.781; *n* = 179). The levels of agreement were significantly higher among aEEG reviewers compared to EEG reviewers (ΔICC = 0.207, 95% CI: 0.149–0.288, *n* = 179). EEG reviewers had systematic differences in interpretation that were not apparent in aEEG review (a significant difference in the average EEG age with respect to reviewer: *P* < 0.001; no significant differences in the average aEEG age with respect to reviewer: *P* = 0.10, Kruskal–Wallis test, *n* = 179). Pairwise correlations between reviewers of the EEG and aEEG are shown in Table 1.

**Table 1:** The correlations between age assessment among the four EEG (left hand side of table) and three aEEG reviewers (right hand side of table). R refers to each reviewer, values given are the correlation coefficient and *range* (CI 95%).

EEG				aEEG		
	R2_EEG_	R3_EEG_	R4_EEG_		R2_aEEG_	R3_aEEG_
R1_EEG_	0.532 *(0*.*379–0*.*638)*	0.724 *(0*.*604–0*.*797)*	0.616 *(0*.*491–0*.*736)*	R1_aEEG_	0.800 *(0*.*753–0*.*844)*	0.722 *(0*.*675–0*.*778)*
R2_EEG_		0.615 *(0*.*500–0*.*689)*	0.721 *(0*.*647–0*.*781)*	R2_aEEG_		0.749 *(0*.*700–0*.*803)*
R3_EEG_			0.687 *(0*.*597–0*.*768)*			

### Correlation of visual review with PMA

**Figure 2:**
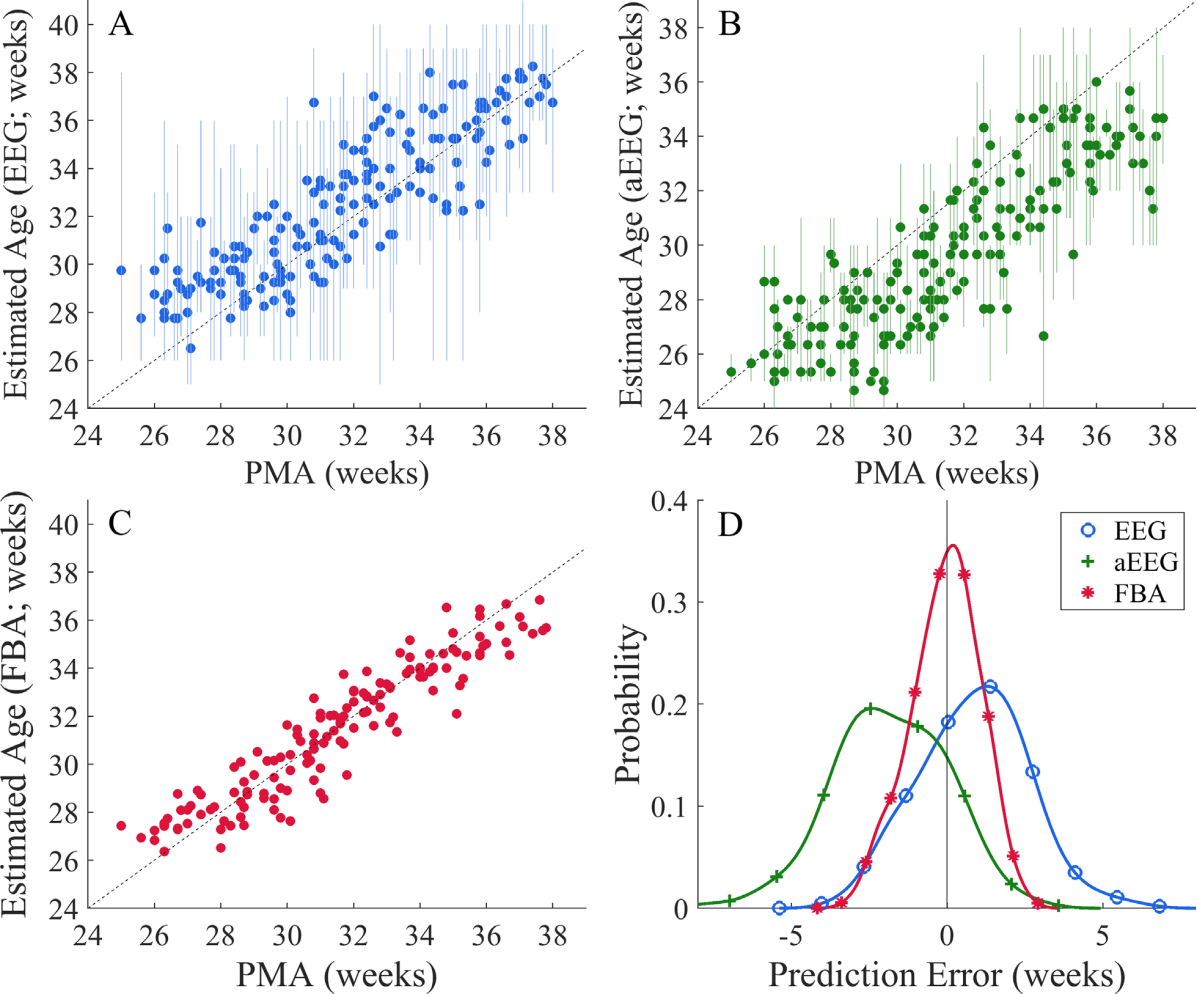
Accuracy of age estimates in the visual EEG (A) and aEEG (B) reviews, and the computational FBA (C), *n* = 149. The filled markers in A and B denote the average estimated age across reviewers for each recording and the associated vertical lines denote the range (minimum to maximum) of estimated ages per reviewer. The diagonal dashed line delineates perfect PMA estimation. (D) Overall comparison of the error distributions of each interpretation type and all reviewers, collapsed over the datasets shown in A–C. The distributions are kernel density estimates.

All visual EEG and aEEG interpretations were strongly correlated with PMA (Fig. 2, Table S2). The average systematic error was 0.8 weeks for EEG reviewers and −1.8 weeks for aEEG reviewers. The average random error was 2.5 weeks for EEG reviewers and 2.3 weeks for aEEG reviewers. Individual errors are shown in Table S4.

**Figure 3:**
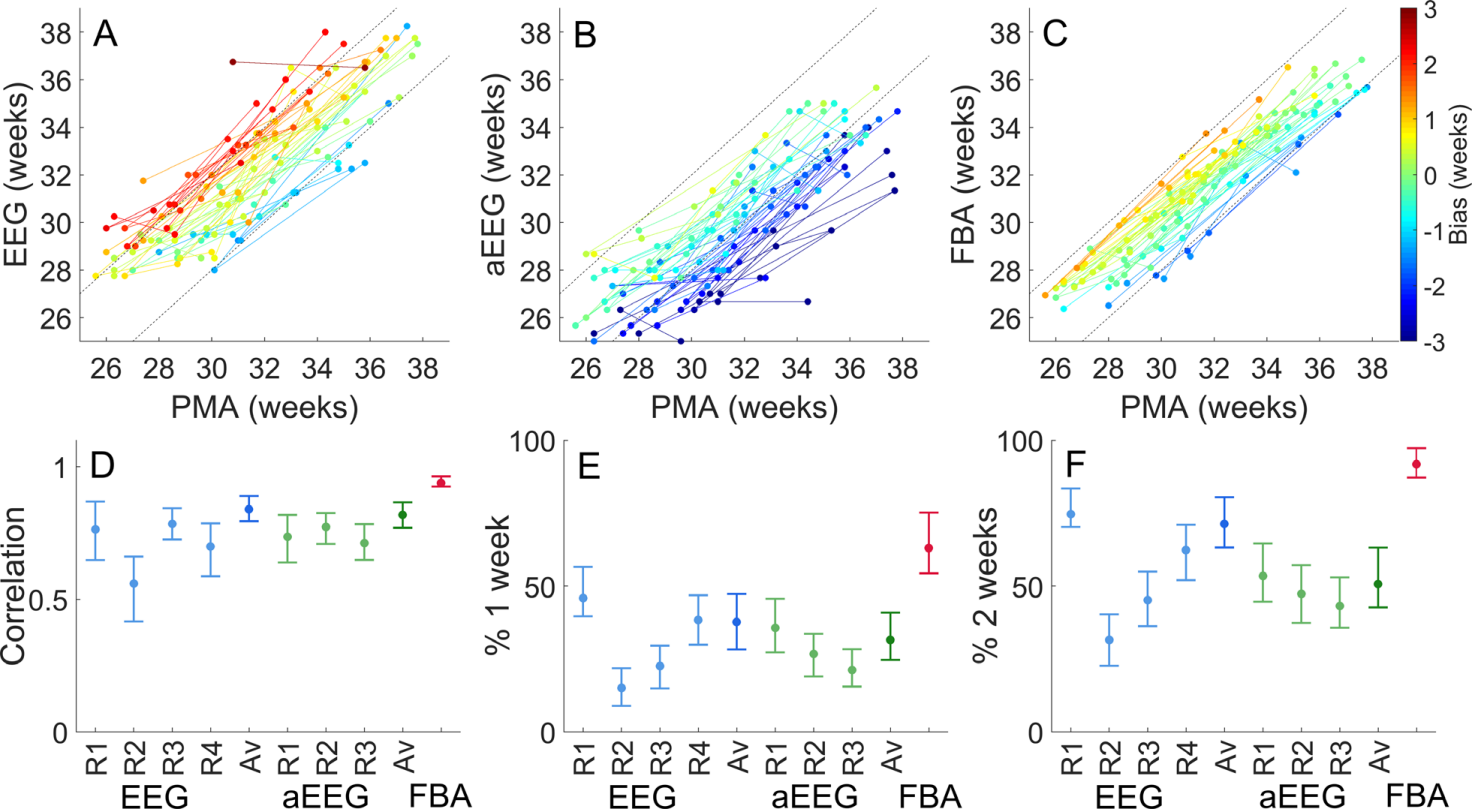
Comparison of age estimates. (A–C) Growth trajectories from infants with serial recordings (*n* = 47). (A) The visual interpretation of the EEG, (B) the visual interpretation of the aEEG and (C) the FBA versus PMA. Trajectories are colored according to the predicted age difference (lag, or bias between the true age and the FBA estimate). (D–E) Summary of the correlation of the visual interpretation of the EEG/aEEG and the FBA with PMA for individual raters (R1, R2, R3 and R4 for the EEG and R1, R2 and R3 for the aEEG) and the ensemble age estimate (Av). (D) Correlation with PMA, (E) Percentage of recordings within 1 week of PMA, (F) Percentage of recordings within 2 weeks of PMA. All values are shown as actual value (circular marker; EEG (blue), aEEG (green) and FBA (red) are colored) and 95% CI (whiskers).

EEG review yielded 1‐week accuracy in 10–52% of recordings, and 2‐week accuracy in 28–75% of recordings (*n* = 146; Fig. 3E and F). The corresponding values with the aEEG review were lower, with 1‐week accuracy obtained in 17–30% of infants, and 2‐week accuracy in 40–62% of infants (*n* = 146; Fig. 3E and F). The visual interpretations of the EEG and aEEG have comparable random errors (*P* = 0.22, Pitman–Morgan test; *n* = 146. The aEEG interpretation tended to underestimate the PMA by 1.8 weeks (Cohen’s *D* = −1.0, *P* < 0.001, *t*‐test; *n* = 146) and the EEG interpretation tended to overestimate the PMA by 0.8 weeks (Cohen’s *D* = 0.5, *P* < 0.001, *t*‐test; *n* = 146; Fig. 2D). The random error of the ensemble reviewer estimate of age was 1.7 weeks and 1.8 weeks for the EEG and aEEG, respectively (*n* = 146; Fig. 2D). The group average (ensemble) interpretation of the EEG and aEEG readers showed stronger correlation with PMA than any individual reviewer (Fig. 3D).

### Visual versus computational interpretation

These two methods of visual review were then benchmarked against the FBA, a computational assessment of functional brain age (Figs. 2C and 3). Compared to the ensemble visual interpretation of EEG or aEEG, the FBA has a lower random error (FBA = 1.1 weeks, EEG = 1.7 weeks, aEEG = 1.8 weeks, *P* < 0.001 and *P* < 0.001, respectively, Pitman–Morgan test; *n* = 146, Fig. 2D and Table S4), and a lower systematic error than the aEEG interpretation (FBA = −0.1 weeks; EEG = 0.8 weeks; aEEG = −1.8 weeks: FBA vs. aEEG: *t*‐test; *P* < 0.001, FBA vs. EEG: *t*‐test; *P* = 0.46, *n* = 146; Fig. 2D). The visual interpretation of the EEG and aEEG were, generally, more highly correlated with the FBA than the PMA; however these differences were not significant (Δr EEG: 0.030, 95%CI: −0.002–0.069 and Δr aEEG: 0.017, 95%CI: −0.014–0.049; *n* = 146, respectively – see Table S3).

### Accuracy in tracking individual brain maturation

When measuring an infant’s “developmental trajectory” via serial recordings of the EEG (*n* = 47), the *intra‐*infant error, which is predominantly a random error, is the most important factor, as systematic errors should be constant as they are assumed to result from *inter*‐infant differences. The maturational trajectories drawn using visual estimates of the EEG or aEEG were strikingly variable, while FBA trajectories were clearly differentiated and consistent (Fig. 3A–C). The FBA had significantly lower “per infant” random error than the visual interpretation of the EEG and aEEG (FBA = 0.6 ± 0.5 weeks, EEG = 1.2 ± 0.8 weeks, aEEG = 1.3 ± 0.9 weeks: ANOVA; *P* < 0.001; post‐hoc comparisons showed differences between FBA vs. EEG, *P* = 0.001, and FBA vs. aEEG, *P* < 0.001). The random error was not significantly different between the visual interpretation of the EEG and aEEG (*P* = 0.89).

### The effect of abnormal neurology on ICC and PMA estimation

The effects of three different definitions of pathologic conditions on PMA estimation were examined. The records were first divided into recordings without pathologic annotations (*n* = 97) versus those with (*n* = 49). The records were next divided into recordings with IVH or PVL (*n* = 112) versus those without (*n* = 34). Finally, the records were divided into recordings with abnormal neurodevelopmental outcome (*n* = 97) versus those without (*n* = 30); infants with 19 EEG recordings were lost to follow‐up.

There were no differences in ICC, systematic or random error with respect to annotations of abnormality as defined by the visual interpretation of the aEEG/EEG groups (Table 2). The random error was significantly higher in infants with abnormal neurodevelopmental outcome for EEG and significantly lower for aEEG review (Table 2).

**Table 2:** The influence of abnormality on visual PMA assessment (averaged across experts).

	Visual Interpretation		Brain lesions		Neurodevelopmental outcome	
	Normal (*n* = 97)	Abnormal (*n* = 49)	Normal (*n* = 112)	Abnormal (*n* = 34)	Normal (*n* = 97)	Abnormal (*n* = 30)
PMA	30.9 (3.0)	31.8 (3.1)	31.5 (3.0)	31.2 (3.5)	31.6 (3.2)	31.7 (3.0)
EEG						
ICC	0.427 (0.238–0.603)	0.489 (0.254–0.661)	0.456 (0.258–0.614)	0.552 (0.310–0.737)	0.499 (0.313–0.646)	0.481 (0.193–0.706)
Systematic error	0.7	0.9	0.8	1.0	0.7	0.4
Random error	1.7	1.7	1.8	1.5	**1.5^c^**	**2.2^c^**
aEEG						
ICC	0.713 (0.586–0.815)	0.687 (0.587–0.770)	0.710 (0.625–0.781)	0.699 (0.535–0.822)	0.694 (0.595–0.775)	0.708 (0.536–0.837)
Systematic error	−2.1	−1.7	−2.0	−1.2	−1.7	−2.5
Random error	2.0	1.7	1.8	1.5	**1.9^c^**	**1.2^c^**
FBA						
Systematic error	0.0	−0.1	−0.1	0.1	0.0	−0.5
Random error	1.0	1.1	1.1	0.9	1.0	1.3

Abnormality was defined in three ways (columns, respectively): visual interpretation of abnormal features in the aEEG/EEG, the presence of brain lesions and abnormal neurodevelopmental outcome. PMA is expressed as mean (standard deviation), ICC is expressed as ICC (95%CI), all errors are in weeks. Significant differences at the 0.05 level between normal and abnormal groups are denoted in bold and with superscripts: ^a^confidence interval of differences did not span 0, ^b^unpaired *t*‐test, ^c^Bartlett test. *P*‐values/CIs were corrected for three different groupings using Bonferroni’s method.

## Discussion

This study assessed the inter‐rater agreement of, and accuracy of PMA estimation using exclusively the visual interpretation of EEG/aEEG recordings by internationally recognized experts. We showed moderate agreement between expert interpretation of functional brain maturity from the EEG or aEEG. Interpretations of the EEG and aEEG were highly correlated with PMA at the individual level, and this correlation was increased when the average of several independent experts was observed. There was, nevertheless, high intra‐subject variability in the PMA estimate for visual EEG/aEEG review. Our work extends beyond the clinical tradition with visual EEG reviews, and we show that an automated computational measure of functional brain age may provide a more accurate estimate of brain maturity.

There was moderate agreement among expert EEG and aEEG readers’ estimations of PMA in preterm infants. The visual interpretation of the EEG and aEEG was highly correlated with PMA. As such, the common posit that an experienced reader should be able to assess EEG maturity with an accuracy of 2 weeks was confirmed. The presence of concurrent pathologic conditions did not significantly impair the estimation of PMA using EEG or aEEG, although the variability in the visually determined PMA estimate was altered in infants with respect to neurodevelopmental outcome for both EEG and aEEG review. There was, nevertheless, high intra‐subject variability in the PMA estimates by visual EEG/aEEG review. This brings into question the validity of creating maturation trajectories in individual infants using the visual interpretation of EEG or aEEG alone. In contrast, this study showed that a novel, algorithm‐based, computational measure of FBA provided more accurate estimates of brain maturity from which stable maturational trajectories could be calculated.

Multiple studies have examined the inter‐rater variability of the visual interpretation of neonatal EEG and aEEG. In the study by Massey et al. (2019), there was substantial agreement for the categorization of EEG voltage but poor to inconsistent agreement for the presence of pathological sharp waves.^23^ Clancy et al. (2011), showed a moderate, positive association between grades of EEG and aEEG background, although the distribution of grades was significantly different.^24^ Inter‐rater agreement of seizure detection by experts using EEG was generally high (*κ* = 0.827) but reduced when seizures were infrequent or of short duration.^25^, ^26^ There was significant differences between the visual review of aEEG and EEG for the detection of neonatal seizures;^27^ differences that could be resolved using a joint interpretation.^28^ In seizure detection, the density of EEG electrodes also has some influence on agreement.^29^ In the present study, there was moderate agreement among experts (ICC aEEG = 0.724; ICC EEG = 0.517). PMA estimates based on the aEEG had a higher level of agreement, but were less accurate, compared to PMA estimates based on the EEG. The higher agreement but lower accuracy among aEEG‐based estimates is likely related to the simplicity of the aEEG trend presenting with fewer degrees of freedom for visual interpretation.^30^

The rapid developmental changes in the EEG were evident using a machine learning–based algorithm. The FBA algorithm was, however, trained on the database of EEG recordings used in this study. While the PMA estimates used cross‐validation, somewhat mitigating to problem of overfitting, further validation of the FBA on an independent population showed similar correlations with PMA as individual experts in this study (see Table S2).^13^ This was not a limitation for EEG and aEEG review, where readers had not seen the recording before and were applying a generalized understanding of EEG/aEEG maturation to the visual interpretation.

The conduct of an inter‐rater study of EEG or aEEG assessment is challenged by many issues. The most obvious is that visual interpretation is inherently a subjective, pattern‐recognition task, so it may be influenced by the visual appearance of the review software and the display settings selected by each reader. The study cohort represented a typical population of preterm infants who were clinically stable at the time of EEG recording but were subjected to common adversities of prematurity such as IVH, PVL, chronic lung disease and CNS‐active drugs. These coincident conditions could confound estimates of PMA but would not likely disturb measures of inter‐rater agreement. This study, therefore, can be assumed to offer a lower bound on the accuracy of the clinical estimate of PMA using the visual interpretation of EEG/aEEG. This study used, exclusively, the visual interpretation of EEG/aEEG recordings by internationally recognized experts. This does not reflect typical clinical practice in which readers must judge EEG maturity in a subject with known PMA. A priori knowledge of the PMA would, however, introduce a bias into any assessment of the accuracy or reliability of visual review.

In conclusion, this study shows that tracking of functional brain maturation is possible using EEG and aEEG. However, developmental trajectories may not be sufficiently accurate or stable for clinical or research purposes. Computational measures hold promise for improving developmental tracking and as a biomarker to benchmark novel therapeutic interventions.^31^, ^32^, ^33^

## Conflict of Interest

JAR and SV hold a patent on the burst metrics used, in part, to generate the FBA measure analyzed in this paper. The remaining authors have no conflicts of interest to report.

## Author Contributions

NJS, SV, and AK contributed to study conceptualization; NJS, JAR and SV undertook preliminary investigations, NJS performed the formal analysis; KKS, SV contributed to data collection and curation; EG, EP, RC, KKS, AK, MLT, and SV performed the annotation of the EEG recordings, SV and KKS acquired funding for the study; and all authors contributed to the writing of the paper.

## Supporting information


**Table S1.** Clinical description of the groups used in the study.
**Table S2.** The correlation between the visual interpretation of EEG/aEEG and PMA.
**Table S3.** Agreement between the average visual interpretation of EEG/aEEG, PMA and an automated estimate of EEG functional brain age (FBA).
**Table S4.** The systematic and random error between the visual interpretation of EEG/aEEG and PMA.
**Figure S1.** Estimation of PMA using the visual interpretation of the EEG.
**Figure S2.** Estimation of PMA using the visual interpretation of the EEG.
**Figure S3.** Estimation of PMA using the visual interpretation of the aEEG.
**Figure S4.** Estimation of PMA using the visual interpretation of the aEEG.
