# Supplementary material for: Reliability and accuracy of EEG interpretation for estimating age in preterm infants
Source: Ann Clin Transl Neurol. 2020 Aug 7;7(9):1564–73. doi: 10.1002/acn3.51132 (PMC7480927; doi:10.1002/acn3.51132)
Supplement: Supplementary file 1 — Table S1. Clinical description of the groups used in the study. Table S2. The correlation between the visual interpretation of EEG/aEEG and PMA. Table S3. Agreement between the average visual interpretation of EEG/aEEG, PMA and an automated estimate of EEG functional brain age (FBA). Table S4. The systematic and random error between the visual interpretation of EEG/aEEG and PMA. Figure S1. Estimation of PMA using the visual interpretation of the EEG. Figure S2. Estimation of PMA using the visual interpretation of the EEG. Figure S3. Estimation of PMA using the visual interpretation of the aEEG. Figure S4. Estimation of PMA using the visual interpretation of the aEEG. [file ACN3-7-1564-s001.pdf]

TABLES FOR SUPPORTING INFORMATION

Table S1: Clinical description of the groups used in the study. Outcome was assessed at either 1 or 2y of age using the Bayley Scales of Developmental Outcome examination (Austrian Norms) – the minimum score across cognitive, language and motor outcomes was used to define 3 grades of outcome: normal was a score greater than 85, mildly abnormal was a score between 70 and 85, and abnormal was a score less than 70. * Several recordings met more than one exclusion criteria.

	ICC group	PMA group
infants (n)	62	61
gestational age (weeks)	25.7 ± 1.5	25.7 ± 1.5
birth weight (g)[a]	736 ± 183	736 ± 184
sex (m:f) [a]	31:30	31:29
Conditions\pathology		
intraventricular hemorrhage	13 (I/II = 10, III/IV = 3)	13 (I/II = 10, III/IV = 3)
periventricular leukomalacia	1 (I/II = 1)	1 (I/II = 1)
necrotizing enterocolitis	2	2
chronic lung disease	18	18
Bayles Scales of Infant Development Outcomes		
Normal	20	20
Mild Abnormal	17	17
Abnormal	15	14
Lost to Follow Up	10	10
EEG recordings (n)	179	146
PMA of EEG recording (weeks)	31.6 ± 3.3<br>[min = 25.0; max = 38.0]	31.4 ± 3.1<br>[min = 25.0; max = 37.8]
Medications at EEG recording		
No medication	13	8
Caffeine	152	127
Morphine	5	5
Doxapram	5	4
Anticonvulsants	0	0
Missing data	9	6
Abnormal EEG recordings	54	49
Excluded Recordings*		
Artefact	22	22
Limited Duration	17	10
Missing Data	37	9

Table S2: The correlation between the visual interpretation of EEG/aEEG and PMA. Note, the sample size is *n* = 146, in order to compare correlations to the output of the FBA algorithm.

EEG Reviewer	Correlation, *r* (95%CI)	aEEG Reviewer	Correlation, *r* (95%CI)
$R1_{EEG}$	0.813 (0.715-0.886)	$R1_{aEEG}$	0.750 (0.660-0.836)
$R2_{EEG}$	0.560 (0.409-0.684)	$R2_{aEEG}$	0.793 (0.753-0.835)
$R3_{EEG}$	0.809 (0.761-0.857)	$R3_{aEEG}$	0.722 (0.655-0.789)
$R4_{EEG}$	0.727 (0.627-0.819)		

Table S3: Agreement between the average visual interpretation of EEG/aEEG, PMA and an automated estimate of EEG functional brain age (FBA). Note, the sample size is reduced (*n* = 146) as the FBA algorithm rejected additional EEG recordings from analysis.

	Correlation, *r* (95%CI)	±1 week (%)	±2 weeks (%)
FBA vs PMA	0.938 (0.926-0.963)	63 (54-75)	92 (87-97)
EEG vs FBA	0.870 (0.834-0.912)	42 (34-50)	75 (68-85)
EEG vs PMA	0.840 (0.794-0.889)	38 (28-47)	71 (63-80)
aEEG vs FBA	0.835 (0.789-0.881)	27 (21-36)	54 (46-67)
aEEG vs PMA	0.818 (0.769-0.865)	32 (25-41)	51 (43-63)

Table S4: The systematic and random error between the visual interpretation of EEG/aEEG and PMA. Note, the sample size is *n* = 146, in order to compare correlations to the output of the FBA algorithm.

EEG Reviewer	Systematic (95%CI)	Random (95%CI)	aEEG Reviewer	Systematic (95% CI)	Random (95% CI)
$R1_{EEG}$	0.3 (0.0 to 0.6)	1.9 (1.5-2.3)	$R1_{aEEG}$	-1.6 (-1.9 to -1.2)	2.0 (1.6-2.3)
$R2_{EEG}$	2.4 (1.7 to 2.9)	3.0 (2.6-3.4)	$R2_{aEEG}$	-1.5 (-1.9 to -1.1)	2.2 (2.0-2.5)
$R3_{EEG}$	2.2 (1.8 to 2.6)	2.3 (2.0-2.4)	$R3_{aEEG}$	-2.2 (-2.6 to -1.7)	2.0 (1.8-2.4)
$R4_{EEG}$	-1.3 (-1.9 to 0.9)	2.2 (1.8-2.6)			
R^{AV}_{EEG}	0.8 (0.4 to 1.2)	1.7 (1.5-1.9)	R^{AV}_{aEEG}	-1.8 (-2.2 to -1.4)	1.8 (1.6-2.0)

FIGURES FOR SUPPORTING INFORMATION

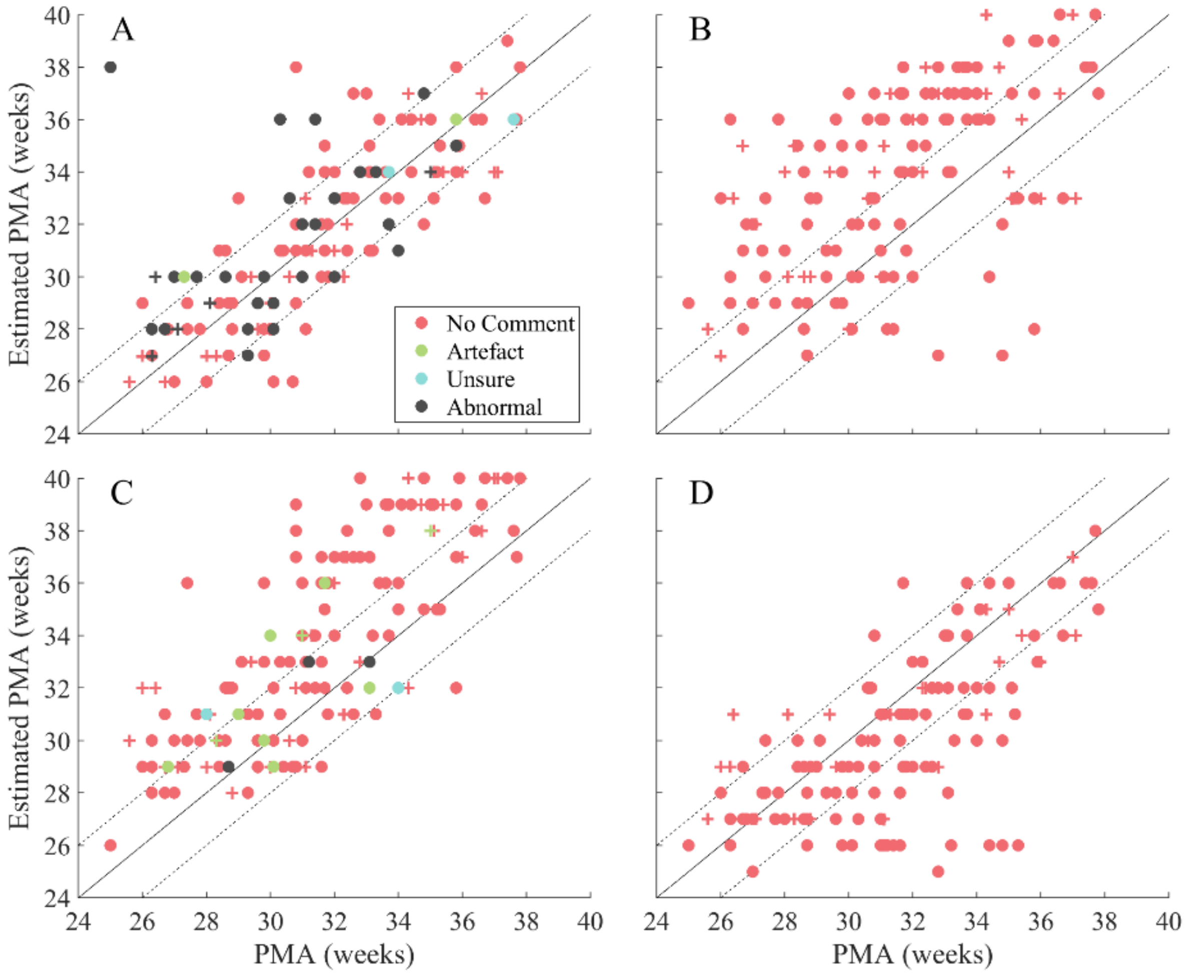

Figure S1: PMA estimated using the visual interpretation of the EEG encoded based on reviewers comments and diagnosis. Estimated PMA is from the visual interpretation of the EEG and PMA is defined as the gestational age plus the post-natal age. A) Reviewer 1, B) Reviewer 2, C) Reviewer 3, D) Reviewer 4. Markers denote a diagnosis of a brain lesion – o denotes recording from infants without IVH/PVL, + denotes recordings from infants with IVH/PVL.

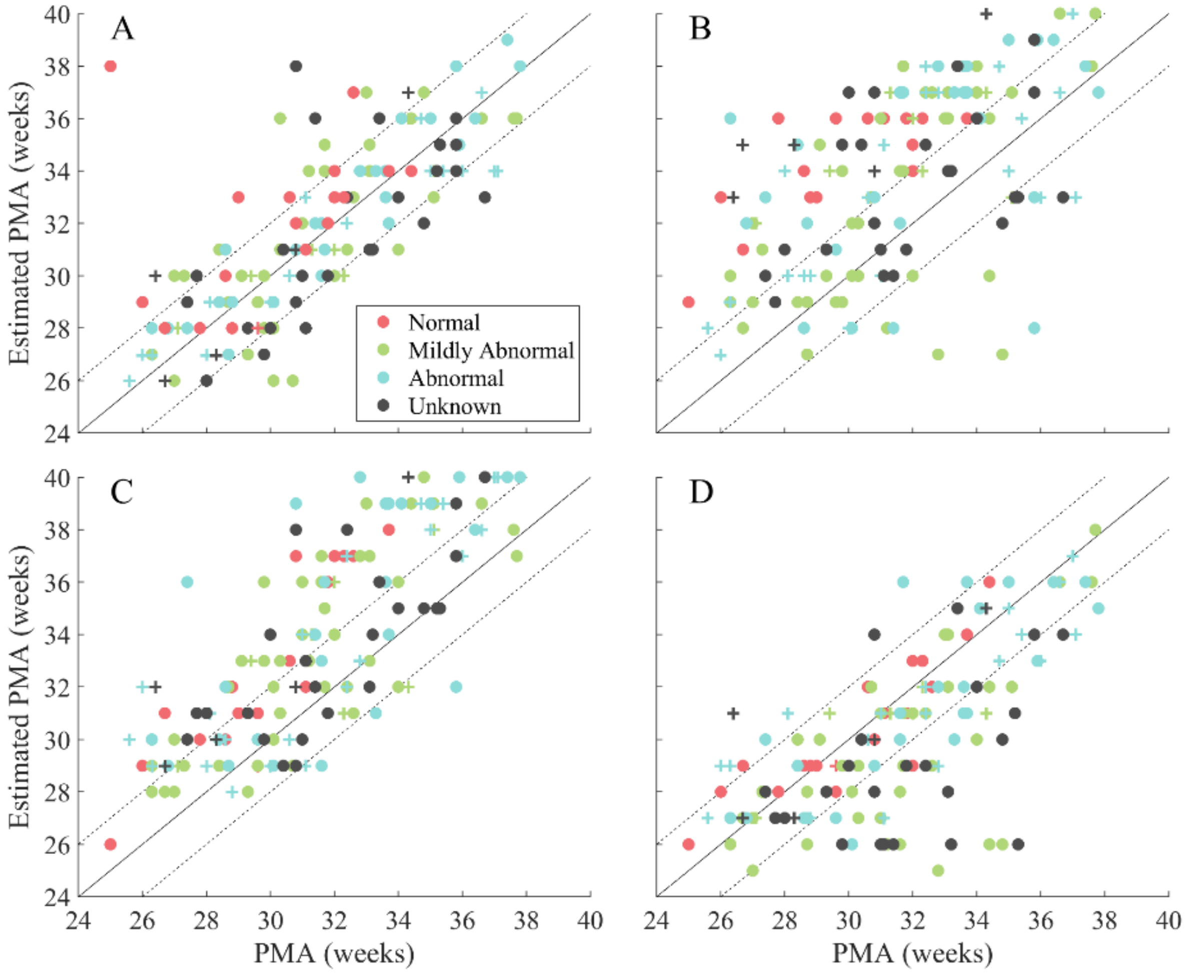

Figure S2: PMA estimated using the visual interpretation of the EEG encoded based on neurodevelopmental outcome and diagnosis. Estimated PMA is from the visual interpretation of the EEG and PMA is defined as the gestational age plus the post-natal age. A) Reviewer 1, B) Reviewer 2, C) Reviewer 3, D) Reviewer 4. Markers denote a diagnosis of a brain lesion – o denotes recording from infants without IVH/PVL, + denoted recordings from infants with IVH/PVL.

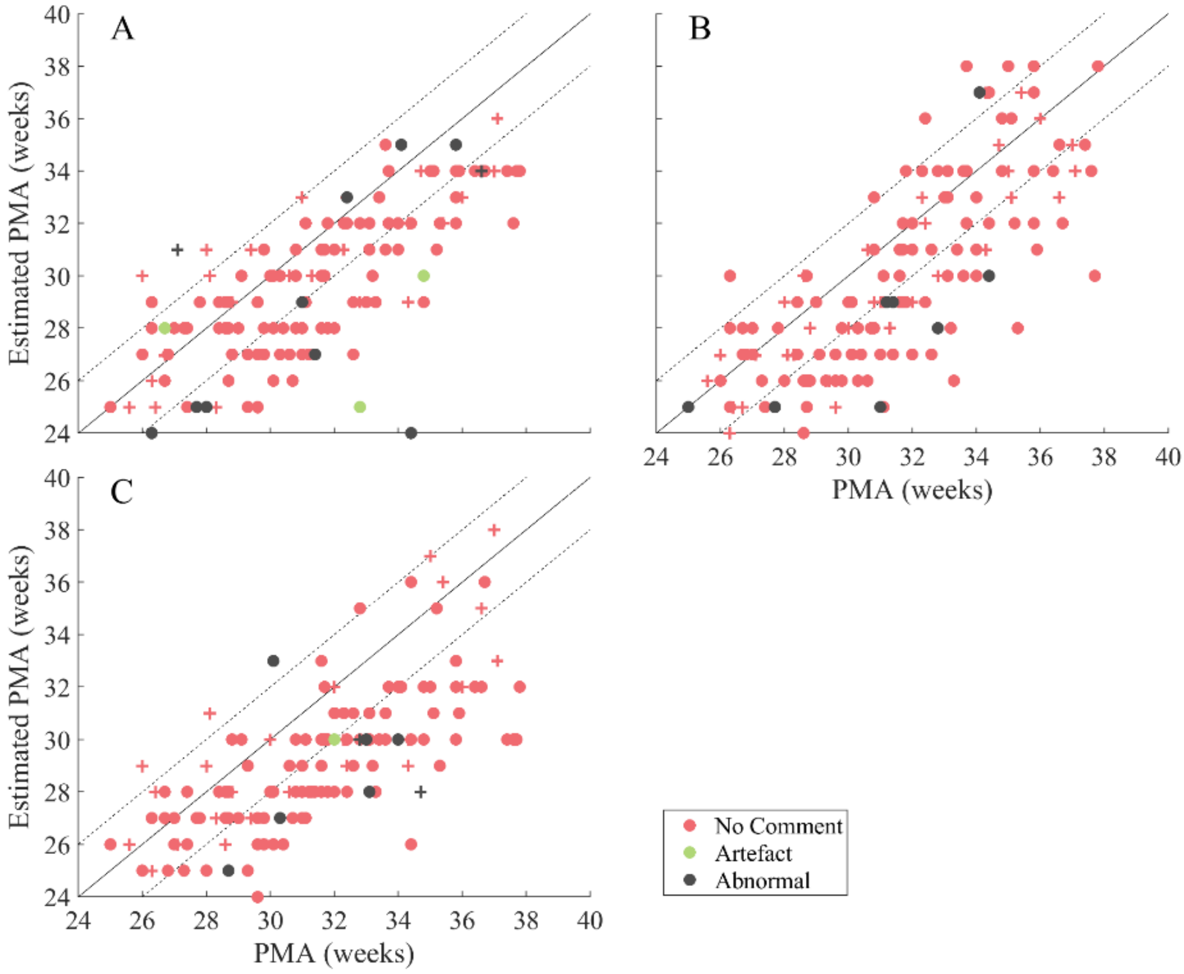

Figure S3: PMA estimated using the visual interpretation of the aEEG encoded based on reviewers comments and diagnosis. Estimated PMA is from the visual interpretation of the aEEG and PMA is defined as the gestational age plus the post-natal age. A) Reviewer 1, B) Reviewer 2, C) Reviewer 3. Markers denote a diagnosis of a brain lesion – o denotes recording from infants without IVH/PVL, + denotes recordings from infants with IVH/PVL.

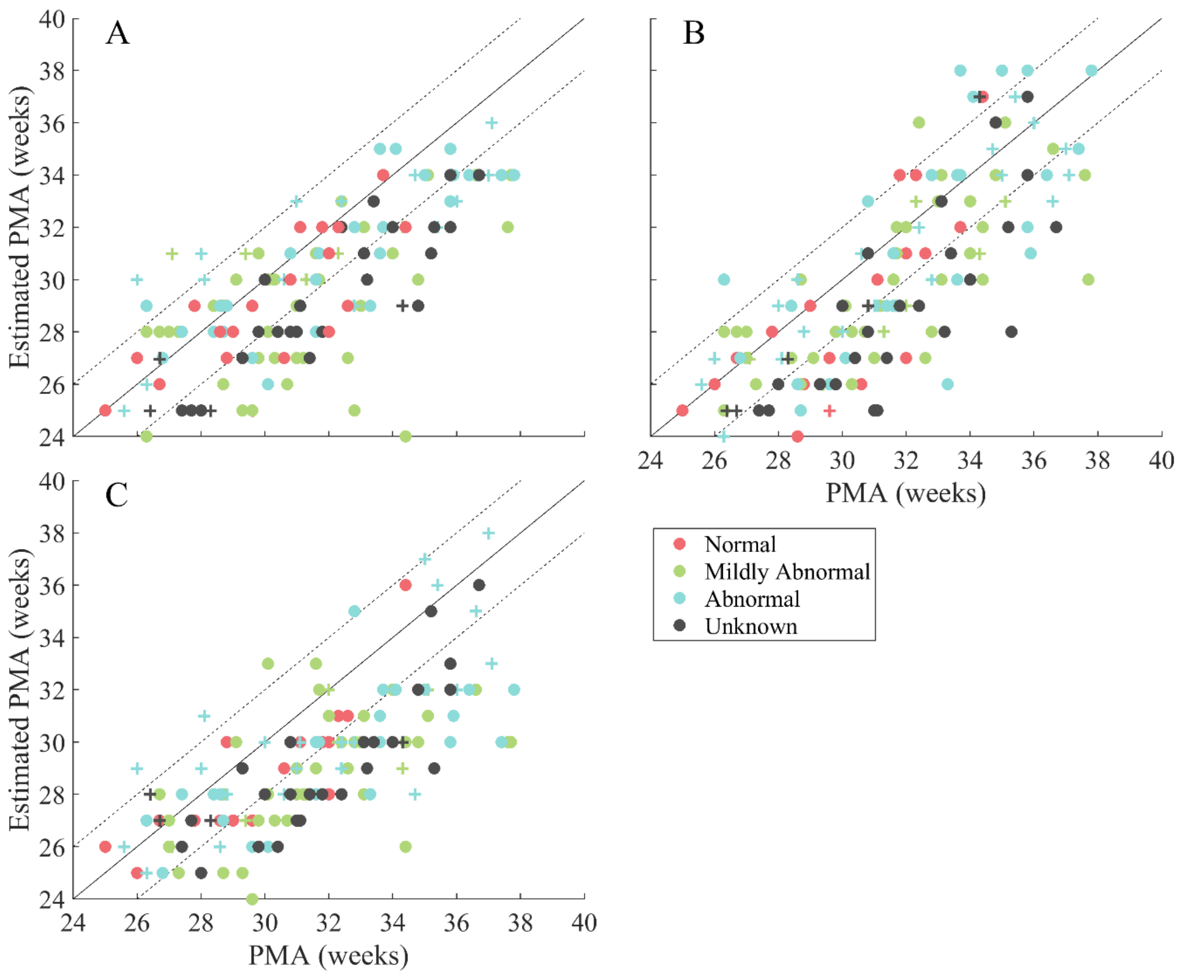

Figure S4: PMA estimated using the visual interpretation of the aEEG encoded based on neurodevelopmental outcome and diagnosis. Estimated PMA is from the visual interpretation of the aEEG and PMA is defined as the gestational age plus the post-natal age. A) Reviewer 1, B) Reviewer 2, C) Reviewer 3. Markers denote a diagnosis of a brain lesion – o denotes recording from infants without IVH/PVL, + denotes recordings from infants with IVH/PVL.
